# Causal relationship between basal metabolic rate and kidney function: a bidirectional two-sample mendelian randomization study

**DOI:** 10.3389/fendo.2024.1319753

**Published:** 2024-04-25

**Authors:** Chaomin Zhou, Yanzhe Peng, Lin Zhan, Yan Zha

**Affiliations:** ^1^ National Health Commission (NHC) Key Laboratory of Pulmonary Immune-related Diseases, Renal Division, Department of Nephrology, Guizhou Provincial People’s Hospital, Guiyang, China; ^2^ GuiZhou University, Medical College, Guiyang, China; ^3^ Research Laboratory Center, Guizhou Provincial People’s Hospital, Guiyang, China

**Keywords:** basal metabolic rate, two-sample mendelian randomization analysis, causal relationship, kidney function, bidirectional

## Abstract

**Background:**

The relationship between basal metabolic rate (BMR) and Chronic kidney disease (CKD) remains unclear and controversial. In this study, we investigated the causal role of BMR in renal injury, and inversely, whether altered renal function causes changes in BMR.

**Methods:**

In this two-sample mendelian randomization (MR) study, Genetic data were accessed from published genome-wide association studies (GWAS) for BMR ((n = 454,874) and indices of renal function, i.e. estimated glomerular filtration rate (eGFR) based on creatinine (n =1, 004, 040), CKD (n=480, 698), and blood urea nitrogen (BUN) (n =852, 678) in European. The inverse variance weighted (IVW) random-effects MR method serves as the main analysis, accompanied by several sensitivity MR analyses. We also performed a reverse MR to explore the causal effects of the above indices of renal function on the BMR.

**Results:**

We found that genetically predicted BMR was negatively related to eGFR, (β= −0.032, P = 4.95*10^-12^). Similar results were obtained using the MR-Egger (β= −0.040, P = 0.002), weighted median (β= −0.04, P= 5.35×10^-11^) and weighted mode method (β= −0.05, P=9.92×10^-7^). Higher BMR had a causal effect on an increased risk of CKD (OR =1.36, 95% CI = 1.11-1.66, P =0.003). In reverse MR, lower eGFR was related to higher BMR (β= −0.64, P = 2.32×10^-6^, IVW analysis). Bidirectional MR supports no causal association was observed between BMR and BUN. Sensitivity analyses confirmed these findings, indicating the robustness of the results.

**Conclusion:**

Genetically predicted high BMR is associated with impaired kidney function. Conversely, genetically predicted decreased eGFR is associated with higher BMR.

## 1 Introduction

Chronic kidney disease (CKD) has become one of the major global public health problems (1). In 2017, the worldwide prevalence of CKD was 9.1%. 1.2 million people died from CKD worldwide in 2017. The global all-age mortality rate has increased by 41.5% from 1990 to 2017 (2). It has been one of the fastest-growing causes of death worldwide and is expected to become the second most common cause of death within the next century in some countries (3, 4). CKD not only causes a high mortality rate but also carries a significant economic burden. In 2020, the United States Renal Data System estimated that healthcare expenditures for CKD patients exceeded $85.4 billion, representing 23.5% of total healthcare expenditures (5). Besides increasing the risk of mortality and economic burden, CKD patients are usually affected by frailty (6), functional and cognitive impairment, reduced quality of life, and disability (7–9). Therefore, early screening for modifiable CKD risk factors to prevent or delay deterioration in kidney function is of great importance.

Major known risk factors for CKD include obesity, hypertension, diabetes, and metabolic abnormalities (10). All of the known risk factors listed above are associated with an abnormal basal metabolic rate (BMR) (11, 12). In an earlier study, BMR had been suggested to be lower in patients with worse renal function (13), although a recent study did not find a direct relationship between reduced kidney function and metabolic rate (14). It is unknown whether BMR might have an impact on susceptibility to kidney injury/CKD and whether a genetically predicted risk of CKD/kidney injury may influence the level of BMR (i.e., reverse causation).

Mendelian randomization (MR) is an emerging method in genetic epidemiology, which has allowed for the inference of causality in the putative exposure-outcome pathway. This approach can effectively overcome the shortcomings of traditional epidemiological studies, such as confounders, reverse causation, and selection bias. To further identify the potential causal effect of BMR on the incidence of CKD/renal injury, we conducted a bidirectional two-sample MR analysis to explain the relationship from a genetic perspective.

## 2 Materials and methods

### 2.1 Study design

**Figure 1:**
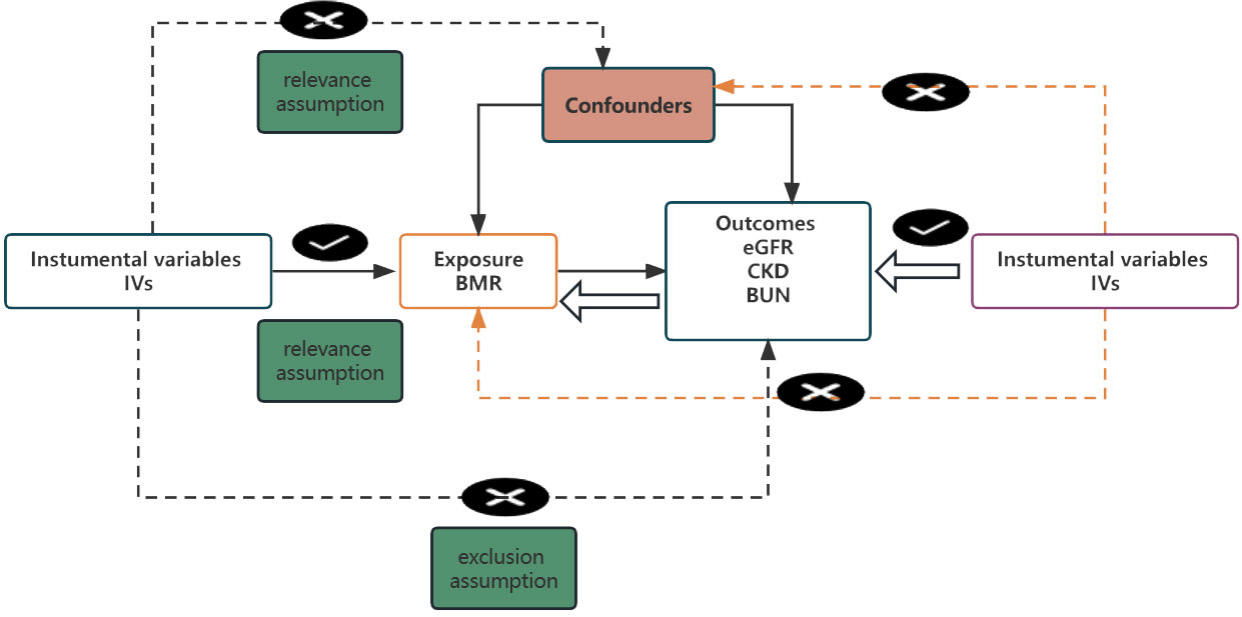
Study flame chart of the al two-sample Mendelian randomization study.

We conducted a 2 sample MR analysis to investigate the causal effect of BMR on renal injury using GWAS summary statistics. **Figure 1** shows a prime description of the bidirectional MR design. The MR analysis emulates the RCT concerning the random assignment of single-nucleotide polymorphisms (SNPs) in the offspring (independent of confounding factors such as sex and age). In addition, this MR design must meet three assumptions: first, that genetic instruments are robustly associated with exposure; second, the association of the instruments with the exposure is independent of the confounders; third, the instruments affect the outcome only through the exposure (15). Ethics approval was not required in our study as all data used were derived from publicly available summary statistics.

### 2.2 Data sources and SNP selection for BMR

The summary-level data for BMR was obtained from a large GWAS database (ID: ukb-b-16446), which included 454,874 cases of European ancestry. Given the MR design in our study, the SNPs should meet the following 3 main assumptions. First, relevance to the exposure. SNPs, associated with BMR were extracted as instrumental variables for corresponding BMR-related traits at the genomewide significance level (P < 5×10^−8^). This approach ensures that genetic variation can effectively serve as a proxy for BMR exposure. Second, independence from confounding factors. SNPs with significant linkage disequilibrium with the measured SNPs (r^2 =^ 0.001) were therefore removed in the range of 10,000 kb. Third, effect on outcomes through the exposure. The selected SNPs were further filtered in the Phenoscanner database, a platform with comprehensive information on the association of genotype and phenotype, to ensure that the included instrumental variables were not correlated with confounding factors (16). To avoid weak instrument bias, only SNPs with an F-statistic greater than 10 were included in our study (17).

### 2.3 Data sources for kidney function

Renal function was assessed using three sets of parameters: estimated glomerular filtration rate (eGFR) based on creatinine, CKD, and blood urea nitrogen (BUN). eGFR (N=1, 004, 040) and BUN (N=852, 678) were obtained from a meta-analysis of the UK Biobank. The summary CKD data were obtained from CKDGen Consortium, the largest meta-analysis of renal function GWAS to date for European ancestry participants, which consisted of 23 cohorts of European ancestry (n = 480, 698; 41, 395 patients and 439, 303 controls) (18). CKD was defined as eGFR of < 60 mL/min/1.73 m^2^ in the present study. All GWAS data used in our study were from individuals of European ancestry. The details of the participant characteristics of the CKDGen Consortium studies have been reported by Wuttke and Stanzick et al. (18, 19).

### 2.4 Reverse MR analyses of the effect of eGFR, CKD and BUN on the BMR

We also explored whether renal injury affects BMR. Thus, we reversed the exposure and outcome inputs and performed a bidirectional MR analysis to determine the impact of kidney injury on BMR. We extracted independent SNPs significantly associated with eGFR, CKD and BUN in the aforementioned large GWAS database and applied the same MR analyses and sensitivity analyses.

### 2.5 MR analysis

Four different methods including inverse-variance weighted (IVW) (20), weighted median (21), MR-Egger (22), and weighted mode were performed. Among these four approaches, the IVW was used as the primary MR effect estimate as it obtains unbiased estimates of the status without horizontal pleiotropy whereas MR-Egger, weighted median, and weighted mode were used as complements to the IVW as they could provide more robust estimates over a wider range of scenarios but were less efficient. We considered a causal relationship between exposure and outcome to exist if the result was significant in the IVW model. We also used the MR-Egger model, weighted median model, and weighted mode model as references, and if positive findings were replicated in these models, we considered them more robust.

### 2.6 Sensitivity analysis

Sensitivity analysis is pivotal in MR studies to detect underlying pleiotropy and heterogeneity. Cochran’s Q statistic, funnel pot, leave-one-out (LOO) analyses and MR-Egger intercept tests were further conducted to detect the presence of pleiotropy and assess the robustness of the results. In particular, a Cochran Q-value of P < 0.05 was taken as an indication of heterogeneity and an MR-Egger test of the intercept of p < 0.05 shows evidence of directional pleiotropy (23). The MR-PRESSO method is also used to assess and correct for horizontal pleiotropy as it has less bias and better accuracy than the IVW method, MR-Egger when the percent of variants with horizontal pleiotropy is below 10% (24).

### 2.7 Statistical analysis

All analyses were performed in R software version 4.3.0. Analyses were implemented by the package TwoSampleMR (version 7.6.2) and MRPRESSO (version 1.0) in R (version 4.3.0). Considering multiple testing (3 tested outcomes: eGFR, BUN and CKD), we also use the Bonferroni corrected P value for significance. The MR P value threshold for significance was set to 0.05/3 = 0.017.

## 3 Results

### 3.1 Causal effect of BMR on reduced eGFR

We identified 546 independent genome-wide significant SNPs for BMR, 67 of which were available in the CKDGen GWAS. After removing SNPs that were significantly associated with the outcomes (p < 5 × 10^-5^) and being palindromic with intermediate allele frequencies, 56 SNPs were remaining for use in the MR analyses. Hypertension, obesity and diabetes are associated with an abnormal basal metabolic rate and they are also well-accepted risk factors for CKD (25, 26). They may be potential confounding factors between BMR and kidney injury. 6 SNPs were filtered by the PhenoScanner database as they correlated with confounding factors including obesity, hypertension and diabetes. The F-statistics range from 11.56 to 152.78. SNPs with potential pleiotropy identified with the MR-PRESSO approach were further removed. Finally, we included 49 instrumental variants for the MR analysis of BMR with eGFR.

As shown in **Table 1**, the result of IVW unveiled a negative effect of BMR on eGFR (β= −0.032, P = 4.95×10^-12^). Similar results were gained using the MR-Egger (β= −0.040, P = 0.002), weighted median (β= −0.04, P= 5.35×10^-11^) and weighted mode approaches (β= −0.05, P=9.92×10^-7^). Steiger directionality test showed that all instrumental SNPs for BMR were stronger predictors of BMR than eGFR, suggesting the causal direction of our analysis.

**Table 1:** MR analyses of the effect of BMR on eGFR, CKD and BUN.

Exposure	Outcome	MR method	B/OR	se	Lo-CI	Up-CI	p-value
BMR	eGFR	IVW	-0.03	0.005	-0.04	-0.02	**4.95E-12**
		MR Egger	-0.04	0.012	-0.06	-0.02	**0.002**
		Weighted median	-0.04	0.006	-0.05	-0.03	**5.35E-11**
		Weighted mode	-0.04	0.008	-0.06	-0.03	**9.92E-07**
BMR	CKD	IVW	1.36	0.103	1.11	1.66	**0.003**
		MR Egger	1.45	0.235	0.91	2.30	0.119
		Weighted median	1.39	0.1615	1.01	1.90	0.042
		Weighted mode	1.84	0.313	1.00	3.40	0.056
BMR	BUN	IVW	0.01	0.0071	-0.01	0.02	0.31
		MR Egger	-0.01	0.018	-0.04	0.03	0.71
		Weighted median	0.01	0.009	-0.01	0.03	0.27
		Weighted mode	0.02	0.016	-0.01	0.053	0.19

eGFR, estimated glomerular filtration rate; BUN, blood urea nitrogen; CKD, chronic kidney disease; BMR, basal metabolic rate; IVW, Inverse variance weighted (random).

Significant P-values are denoted in bold.

**Figure 2:**
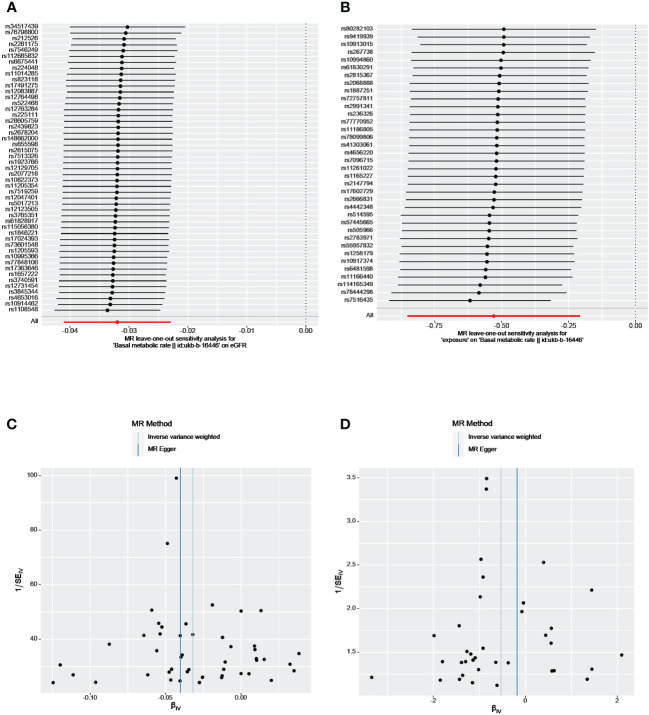
The leave-one-out analyses and funnel plots from exposure on outcome. **(A, C)** From BMR on eGFR; **(B, D)** From eGFR on BMR. eGFR, estimated glomerular filtration rate; BMR, basal metabolic rate.

As shown in **Table 2**, the P value of the MR-Egger intercept between BMR and eGFR was nonsignificant (P=0.46), implying the absence of unbalanced horizontal pleiotropy in our instruments. The IVW and MR Egger Q statistics suggested significant heterogeneity in the estimates (Q = 74.23, p = 0.009 and Q = 73.38, p=0.008). The leave-one-out analysis and funnel plot also revealed that the results were robust (**Figures 2A, C**).

**Table 2:** Tests of heterogeneity, pleiotropy and MR-Steiger causal direction.

Exposure	Outcome	Heterogeneity test			MR-Egger intercept	p- value	MR-Steiger causal direction
		MR method	Q	Q_p- value			
BMR	eGFR	MR-Egger	73.38	0.008	0.0001	0.46	True
		IVW	74.23	0.009			
BMR	CKD	MR-Egger	70.46	0.270	-0.001	0.76	True
		IVW	70.56	0.300			
BMR	BUN	MR-Egger	100.54	0.003	0.0002	0.41	True
		IVW	101.60	0.003			
eGFR	BMR	MR-Egger	60.70	0.001	-0.0003	0.71	True
		IVW	60.98	0.002			
CKD	BMR	MR-Egger	–	–			
		IVW	1.75	0.19	–		
BUN	BMR	MR-Egger	28.10	0.014	-0.002	0.27	True
		IVW	30.75	0.009			

eGFR, estimated glomerular filtration rate; BUN, blood urea nitrogen; CKD, chronic kidney disease; BMR, basal metabolic rate; IVW, Inverse variance weighted (random).

Significant P-values are denoted in bold.

### 3.2 Causal effect of BMR on CKD

After harmonization, 66 genetic variants were available in both exposure and outcome datasets. All the extracted SNPs passed the MR-Steiger filtering. The F-statistics range from 11.56 to 176.59 (mean 32.40). The result of IVW indicated strong evidence that higher BMR had a causal effect on an increased risk of CKD (OR =1.36, 95% CI = 1.11-1.66, p =0.003). The direction of effects was mainly consistent across different methods. As shown in **Table 2**, no horizontal pleiotropy existed between BMR and CKD as the P value of the MR-Egger intercept tests was > 0.05 in our study. There was no heterogeneity in our study as well (Q = 70.46, P = 0.27). The MR-PRESSO did not detect any outliers. Moreover, the leave-one-out analysis revealed that no SNP drove the results, and funnel plots were symmetrical (**Supplementary Figures 1A, B**) indicating that neither estimate was violated.

### 3.3 Causal effect of BMR on BUN

Of the 546 BMR-associated SNPs, 74 were available from the CKDGen GWAS. After harmonization, 6 SNPs were excluded for being palindromic with intermediate allele frequencies from BMR. One of the SNPs with potential pleiotropy identified using the MR-PRESSO approach was excluded further. Therefore, we included 67 SNPs in the MR analysis. No causal association was detected between genetic BMR and BUN (**Table 1**). No horizontal pleiotropy was identified by the MR-Egger test. No heterogeneity was observed between BMR and BUN (IVW analysis: Q = 70.56, P=0.29) (**Table 2**).

### 3.4 Effects of eGFR, CKD and BUN on BMR

We identified 47, 2 and 24 independent genome-wide significant SNPs for eGFR, CKD, and BUN, respectively. After applying harmonization and MR-PRESSO, 33 index SNPs were selected to genetically predict BMR and 16 index SNPs were used to genetically predict BUN. Since only two SNPs were found to be significantly and independently associated with CKD, the MR-PRESSO method was not applied. Overall, all selected SNPs displayed F-statistic >10. Our reverse analysis showed strong evidence of a negative effect of genetically determined eGFR on BMR(β= −0.64, P = 2.32×10^-6^, IVW analysis) (**Table 3**). Similar results were gained using the weighted median (β= −0.85, P= 3.45×10^-8^) and weighted mode approaches (β= −0.95, P=4.34×10^-5^). The results from MR-Egger indicated a nonsignificant but consistent direction. No horizontal pleiotropy existed as the P value of the MR-Egger intercept test was > 0.05 (**Table 2**). The leave-one-out analysis also revealed that no single SNP drove the MR estimates, and the funnel plots were symmetrical, indicating that the results were robust (**Figures 2B, D**). MR Steiger test identified no evidence of reverse causality, and the causal direction was reliable. However, heterogeneity was observed in the Q-test analysis between eGFR and BMR (**Table 2**). There was no significant association between genetic liability and CKD, BUN and BMR (all p > 0.05). Heterogeneity was observed between BUN and BMR with a Cochran Q-test derived p-value of 0.01 of MR-Egger and p-value of 0.009 of IVW. No horizontal pleiotropy existed between BUN and BMR.

**Table 3:** Reverse MR analyses of the effect of eGFR, CKD and BUN on BMR.

Exposure	Outcome	MR method	B/OR	se	Lo-CI	Up-CI	p-value
eGFR	BMR	IVW	-0.64	0.13	-0.90	-0.37	**2.32E-06**
		MR Egger	-0.53	0.31	-1.14	0.08	0.10
		Weighted median	-0.85	0.15	-1.15	-0.55	**3.45E-08**
		Weighted mode	-0.95	0.20	-1.34	-0.56	**4.34E-05**
CKD	BMR	IVW	1.01	0.02	-0.03	0.05	0.60
		MR Egger	–	–	–	–	–
		Weighted median	–	–	–	–	–
		Weighted mode	–	–	–	–	–
BUN	BMR	IVW	0.21	0.14	-0.01	0.02	0.31
		MR Egger	0.74	0.48	-0.04	0.03	0.71
		Weighted median	0.19	0.15	-0.01	0.03	0.27
		Weighted mode	-0.02	0.29	-0.01	0.05	0.19

eGFR, estimated glomerular filtration rate; BUN, blood urea nitrogen; CKD, chronic kidney disease; BMR, basal metabolic rate; IVW, Inverse variance weighted (random).

Significant P-values are denoted in bold.

## 4 Discussion

We conducted a bi-directional two-sample Mendelian randomized study, which could preclude confounding factors and identify causal determinants of a certain outcome efficiently. To the best of our knowledge, this is the first large-scale MR analysis that has sought to explore the causal association between BMR and eGFR, CKD, and BUN. We find a potential bidirectional negative causal relationship between BMR and eGFR. These relationships were mostly consistent across the different MR methods, suggesting the reliability of our findings.

Data are conflicting concerning the association between BMR and CKD. A small number of studies have assessed BMR/resting energy expenditure (REE) in CKD patients (13, 27, 28). But the results were contradictory. Some found the REE in patients with end-stage kidney disease to be increased (29), whereas others came to the opposite conclusion (13, 30). The major limitation of these studies, is that they are all observational studies that could potentially lead to biased associations and conclusions, as they could not entirely rule out the possibility of reverse causality and confounding factors (31). In addition, the sample sizes in these studies are small. Although there was some evidence of heterogeneity in our study, pleiotropy was not detected by Egger’s intercepts, suggesting that no pleiotropic bias was introduced to the MR estimates in the setting of heterogeneity. In addition, the LOO analyses did not reveal any outlier SNPs, and funnel plots were approximately symmetric, indicating that none of the estimates were violated. Our study also supported a potential causal relationship between higher BMR and risk of CKD as IVW yielded P-values less than 0.017 and the directions of MR-Egger regression and Weighted mode approach were consistent with IVW. No heterogeneity and pleiotropy were detected. Our findings provide novel insight into the prevention of CKD and eGFR decline.

Increased BMR appears to be an indicator of perturbations within the metabolic and/or endocrine systems. A higher BMR may indicate metabolic abnormal conditions such as diabetes (32), hypertension (33), obesity and vascular disorders, which may mediate the causal effect of BMR on reduced eGFR or CKD. Patients with diabetes, hypertension and obesity are at an increased risk of CKD and worsening kidney function. Furthermore, a higher rate of metabolism may produce excessive harmful reactive oxygen species. It has been suggested in previous studies that uncontrolled or persistent increases in reactive oxygen species (ROS) can lead to inflammation and fibrosis resulting in kidney injury and CKD progression (34). In addition, a higher BMR is also associated with higher systemic inflammation, which plays a key role in the etiology, progression, and pathophysiology of CKD (35).

In agreement with previous observational studies (29, 36), our findings of inverse MR suggested that lower eGFR was related to higher BMR. The mechanisms are not well understood but several potential mechanisms can be postulated. Patients with decreased eGFR often suffer from increased sympathetic and renin‐angiotensin system (RAS) overactivation or hypertension. A recent study has suggested that the elevated resting metabolic rate (RMR) can be normalized by antagonizing the renin‐angiotensin system in obese or overweight patients with hypertension (37). Thus, the association between decreased eGFR and increased BMR may be attributed to the overactivation of the RAS. On the other hand, the mitochondrial uncoupling proteins (UCPs), which regulate ATP synthesis and the production of reactive oxygen species, may also contribute to the increased BMR in patients with decreased eGFR. Previous studies have suggested that UCP-1 and UCP-3, two key regulators of energy expenditure in humans were increased in uremic status (38, 39). Furthermore, complications associated with worsening kidney function such as increased fluid overload, chronic cardiac failure, chronic anemia, and secondary hyperparathyroidism could lead to an increase in REE (40, 41). Future work is warranted to decipher the underlying mechanisms that link BMR to eGFR.

There are several limitations associated with our study. First, we assessed only genetic liability to BMR and kidney injury, with no regard to the effects of the environment, which are critical for both BMR and kidney function. Second, as this study was conducted among European ancestry participants, the finding may not extend to other ethnic populations. Third, even though we had excluded SNPs that were associated with hypertension, obesity, and diabetes, the causal associations identified in our study could be mediated by other potential confounding factors that could not be entirely excluded.

## 5 Conclusion

In summary, genetically predicted high BMR is associated with impaired kidney function. Conversely, genetically predicted decreased eGFR is associated with higher BMR.

## Data availability statement

All data used in the current study are publicly available GWAS summary data which can be found here: https://gwas.mrcieu.ac.uk/, ID: ukb-b-16446.

## Ethics statement

Ethical approval was not required for the study involving humans in accordance with the local legislation and institutional requirements. Written informed consent to participate in this study was not required from the participants or the participants’ legal guardians/next of kin in accordance with the national legislation and the institutional requirements.

## Author contributions

CZ: Writing – original draft, Writing – review & editing. YP: Data curation, Formal analysis, Investigation, Software, Writing – review & editing. LZ: Methodology, Project administration, Resources, Visualization, Writing – review & editing. YZ: Data curation, Funding acquisition, Resources, Software, Writing – review & editing.
