# Supplementary figures and images for: Causal relationship between basal metabolic rate and kidney function: a bidirectional two-sample mendelian randomization study
Source: Front Endocrinol (Lausanne). 2024 Apr 25;15:1319753. doi: 10.3389/fendo.2024.1319753 (PMC11079271; doi:10.3389/fendo.2024.1319753)

Supplementary Fig.S1
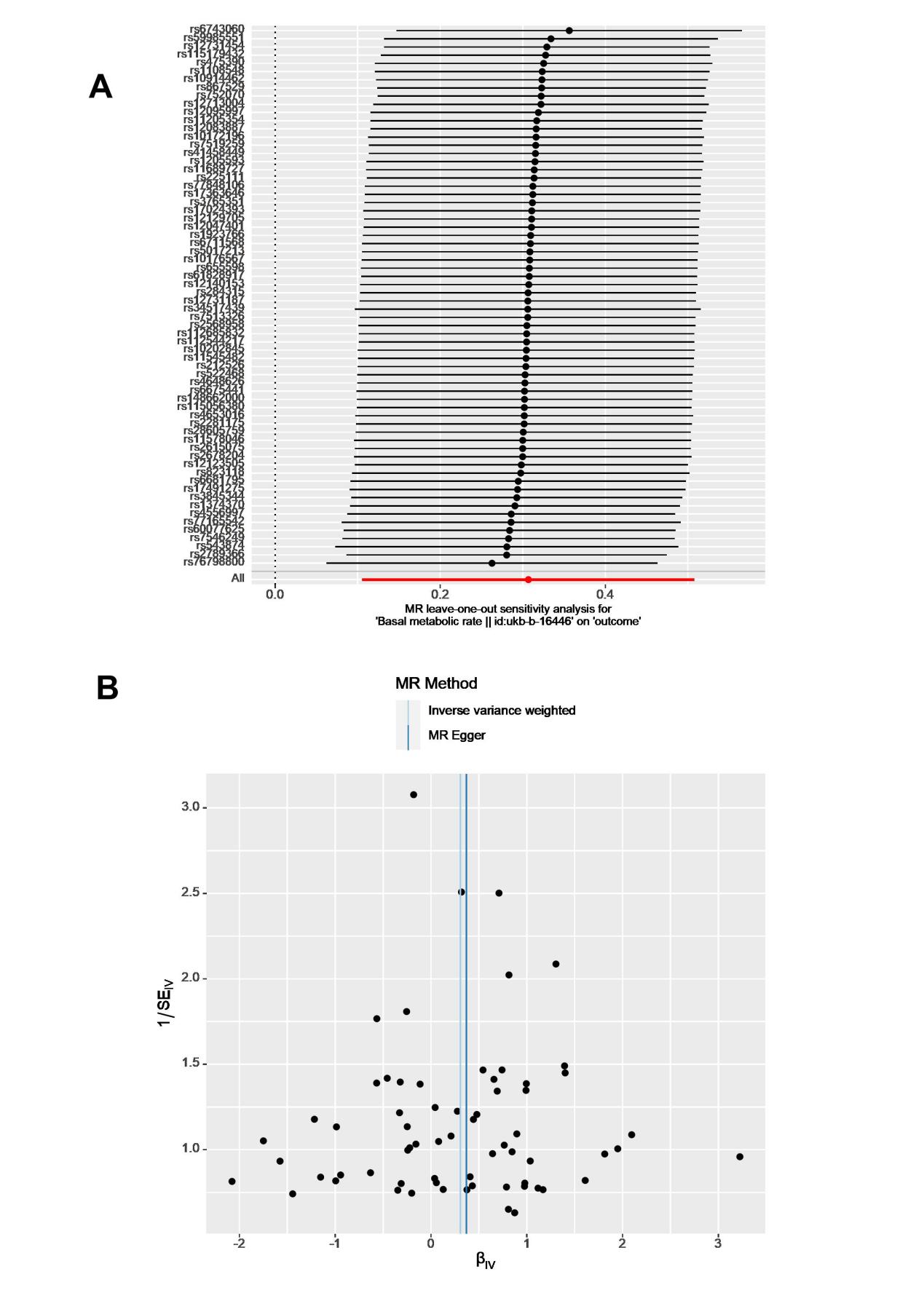

Supplement: Supplementary file 1 [file DataSheet_1.docx]
